# Factors Associated with Nursing Activities in Humanitarian Aid and Disaster Relief

**DOI:** 10.1371/journal.pone.0151170

**Published:** 2016-03-09

**Authors:** Norihito Noguchi, Satoshi Inoue, Chisato Shimanoe, Kaoru Shibayama, Koichi Shinchi

**Affiliations:** 1 Department of Community and International Health Nursing, Faculty of Medicine, Saga University, Saga, Japan; 2 Department of Emergency Medicine, Division of Trauma Surgery and Surgical Critical Care, Faculty of Medicine, Saga University, Saga, Japan; 3 Department of Preventive Medicine, Faculty of Medicine, Saga University, Saga, Japan; 4 Department of Nursing, Saga-Ken Medical Centre Koseikan, Saga, Japan; University of Reading, UNITED KINGDOM

## Abstract

**Background:**

Although nurses play an important role in humanitarian aid and disaster relief (HA/DR), little is known about the nursing activities that are performed in HA/DR. We aimed to clarify the nursing activities performed by Japanese nurses in HA/DR and to examine the factors associated with the frequency of nursing activities.

**Methods:**

A self-administered questionnaire survey was completed by 147 nurses with HA/DR experience. The survey extracted information on demographic characteristics, past experience (e.g., disaster medical training experience, HA/DR experience), circumstances surrounding their dispatched to HA/DR (e.g., team size, disaster type, post-disaster phase, mission term), and the frequency of nursing activities performed under HA/DR. The frequency of nursing activities was rated on a 5-point Likert scale. Evaluation of nursing activities was conducted based on the “nursing activity score”, which represents the frequency of each nursing activity. Factors related to the nursing activity score were evaluated by multiple logistic regression analysis.

**Results:**

Nurses were involved in 27 nursing activities in HA/DR, 10 of which were performed frequently. On analysis, factors significantly associated with nursing activity score were nursing license as a registered nurse (OR 7.79, 95% CI 2.95–20.57), two or more experiences with disaster medical training (OR 2.90 95%, CI 1.12–7.49) and a post-disaster phase of three weeks or longer (OR 8.77, 95% CI 2.59–29.67).

**Conclusions:**

These results will contribute to the design of evidence-based disaster medical training that improves the quality of nursing activities.

## Introduction

Over the past few decades, the number of disasters and the degree of damage they cause has increased markedly worldwide [1–2]. When a large-scale disaster strikes overseas, foreign field hospitals [3–4] and foreign medical teams (FMTs) [5–6] are dispatched to the disaster area as humanitarian aid and disaster relief (HA/DR) to provide medical care for affected people. The World Health Organization (WHO) defines an FMT as groups of health professionals and supporting staff outside their country of origin who aim to provide health care specifically to disaster-affected populations, including governmental (both civilian and military) and non-governmental teams [7]. WHO has established standards and competencies for FMTs in the event of sudden-onset disasters [7]. To date, however, no clear overall view of the disaster relief activities of FMTs has been available.

Japan’s official FMT providers of HA/DR are the Japan Medical Team for Disaster Relief (JMTDR), dispatched by the Japan International Cooperation Agency, and the Japan Ground Self-Defense Force medical team (JGSDF-MT), dispatched by the Ministry of Defense. The JMTDR provides medical care for victims of natural disasters overseas such as International Disaster Relief Activities [8–9], whereas the JGSDF-MT provides medical care for victims of natural and man-made disasters. Moreover, the JGSDF-MT provides health care for Self-Defense Force personnel engaged in reconstruction of infrastructure in disaster-hit areas [10]. However, few studies have examine medical activities conducted during HA/DR by the JGSDF-MT.

Because nursing professionals develop competence in a wide range of care, from emergency medical services in the acute phase of disaster, to mental health nursing care, infectious disease prevention and health guidance in the mid- and long-term phase [11], disaster nursing is expected to play an important role in the overall response to disaster. As preparation for HA/DR, nursing staff members undertake disaster medical training [11–12] and competency development [13–14]. To our knowledge, however, data on nursing activities and related factors in HA/DR is scarce. We considered that the availability of such data would provide reliable empirical evidence for the development of disaster nursing research and training.

Here, to guide future disaster health professionals training and certification programs, we surveyed nursing staff from the JGSDF-MT who worked in HA/DR regarding the nursing activities they undertook, as well as factors associated with these activities, such as demographic characteristics, past experience, and circumstances surrounding dispatch.

## Methods

### Study Subjects

The study was conducted among 147 nurses who had previous experience with one or more medical support activities in HA/DR with the Japan Ground Self-Defense Force (JGSDF) during the period between 1993 and 2013.

### Data Collection

Prior to the start of the survey, we conducted a pilot study to confirm the validity of the questionnaire in 11 nurses with HA/DR experience. Valid responses were obtained from eight. From the responses, the contents of the questionnaire were revised.

For the survey, we recruited 151 subjects affiliated with eight Self-Defense Force hospitals and 30 medical corps throughout Japan with the support of the Medical Department of the Ground Staff Office of the Ministry of Defense. The survey was conducted between September and December 2013.

Subjects were clearly informed in writing that their participation in the study was voluntary and that their return of the anonymous self-administered questionnaire would be taken as consent to participate in the study. The privacy, confidentiality and rights of the subjects were protected throughout the study. The study was approved by the Ethics Committee of Saga University Faculty of Medicine (approval no. 25–22).

### Measures

#### Nursing activities in HA/DR

Based on previous research [14–16] and the JMTDR training curriculum, 42 nursing activities in HA/DR were selected for use in this study. Briefly, nursing activities were validated by a panel of seven experts (two doctors and five nurses) who specialized in disaster nursing, global health, nursing education, medical education, clinical nursing, infection disease and emergency care. Using a 4-point rating scale (1 = not relevant, 2 = somewhat relevant, 3 = quite relevant, 4 = very relevant), the seven experts gave these 42 nursing activities ratings of 3 or 4, resulting in a scale-content validity index (S-CVI) of 0.91. The item-content validity index (I-CVI) ranged from 0.71 to 1.00. The content validity index is taken as a barometer of item and instrument clarity, homogeneity and relevance. The recommended S-CVI minimum scores for evaluating the overall validity of the instrument is ≥0.9 [17], and an I-CVI value of ≥0.78 is therefore considered acceptable [18]. Overall reliability of the 42 nursing activities by Cronbach’s α coefficient was 0.91. The values indicate high internal consistency.

The subject rated each item using a 5-point Likert scale with the responses “never implemented” (1 point), “rarely implemented” (2 points), “sometimes implemented” (3 points), “frequently implemented” (4 points) and “always implemented” (5 points). Nursing activities were evaluated by the nursing activity score, which reflects the frequency of each nursing activity, to give a total score range of 42 to 210 points. A higher score indicated that the activity was more frequently performed in HA/DR.

#### Factors associated with the nursing activity score

Based on the decision-making model of helping in an emergency [19], we hypothesized that personal variables and situational variables were social factors which influenced the frequency of nursing activities in HA/DR. Personal variables consisted of “demographic characteristics” and “past experience”, i.e., past experience before the recent experience in dispatch for HA/DR. Situational variables were considered as the “dispatch situation”, i.e., the situation at the time they were dispatched for HA/DR. The factors associated with the nursing activity score were established as 10 independent variables covering demographic characteristics (sex, age, nursing license, years of experience in nursing), past experience (disaster medical training experience, HA/DR experience), and dispatch situation (team size, disaster type, post-disaster phase and mission term). “Post-disaster phase” is the duration of time from the occurrence of the disaster to the day of dispatch for HA/DR in weeks.

### Statistical Analysis

Variables for analysis were evaluated with consideration to the relationship between the nursing activity score and any influencing factors. The presence of multicollinearity between the 10 independent variables was confirmed by calculation of Spearman correlation coefficients. The correlation coefficient between “nursing license (registered nurse)” and “sex (female)” was r = 0.87. We considered that the nursing activity score was more strongly influenced by differences in “nursing license” than by those in “sex”, and the excluded “sex”. Next, the correlation coefficient between “age” and “years of experience in nursing” was r = 0.72. Because the meaning of these two variables was similar, “years of experience in nursing” was included as an independent variable, while “age” was excluded. The results also showed a strong correlation between “disaster type”, “post-disaster phase” and “mission term”. We therefore included “post-disaster phase” as an independent variable, and excluded “disaster type” and “mission term” on the basis that only “post-disaster phase” demonstrated a significant correlation with the dependent variables. Independent variables included in the final analysis were “nursing license”, “years of experience in nursing”, “disaster medical training experience”, “HA/DR experience”, “team size” and “post-disaster phase”.

Factors related to the nursing activity score were analysed by multiple logistic regression analysis. The dependent variables were divided into two groups (“High” or “Low”), depending on the median value of the nursing activity score, i.e., “High” was median or more and “Low” was less than median. The independent variables were divided into two groups or three groups and the strength of their relationship to the nursing activity score was compared. The groupings were as follows: sex, male or female; nursing license, registered nurse (RN) or licensed practical nurse (LPN); years of experience in nursing (years), < 10, 11–20, or ≥ 21; disaster medical training experience, none, once, or ≥ twice; HA/DR experience, none, once, or ≥ twice; team size, standard (fewer than 24 health professionals per team) or large (24 or more health professionals per team); disaster type, natural disaster or man-made disaster; post-disaster phase, < 1 week, 1–3 weeks, or ≥ 3 weeks; and mission term, < 1 month or ≥ 1 month.

Statistical significance was set at p<0.05. All analyses were conducted using IBM SPSS Statistics version 21 (IBM Japan, Tokyo, Japan).

## Results

A total of 151 questionnaires were administered and 147 valid responses were obtained (valid response rate, 98.7%). Table 1 shows the subject demographics. Most were male (62%), and about 60% had the LPN nursing license. Significant relationships were seen between sex and nursing license, and nursing activity score. Regarding past experience, significant relationships were seen between disaster medical training experience and HA/DR experience, and nursing activity score. Further, more than half of the respondents had taken part in HA/DR without disaster medical training experience. Regarding dispatch situation, the responses indicated significant relationships between team size and post-disaster phase, and nursing activity score. No significant relationship was seen between nursing activity score and age, years of experience in nursing, disaster type or mission term.

**Table 1:** Characteristics of the study subjects by nursing activity score.

Characteristic		Nursing Activity Score		
		Low^a^ (n = 69)	High^a^ (n = 78)	*p* value^b^
Sex	Male	78.3	47.4	<0.001
	Female	21.7	52.6	
Age (years)	Mean (SD)	38.8 (7.0)	39.6 (8.2)	0.55
	25–34	27.5	30.3	0.78
	35–44	49.3	43.4	
	45–54	23.2	26.3	
Nursing license	RN	21.7	56.4	<0.001
	LPN	78.3	43.6	
Years of experience in nursing (years)	Mean (SD)	14.5 (6.8)	15.6 (8.2)	0.37
	≤ 10	27.9	28.9	0.33
	11–20	51.5	40.8	
	≥ 21	20.6	30.3	
Past experience^c^				
Disaster medical training experience	None	60.3	31.6	<0.001
	Once	17.6	21.1	
	≥ twice	22.1	47.4	
HA/DR experience	None	82.6	51.0	0.02
	Once	14.5	19.2	
	≥ twice	2.9	15.4	
Dispatch situation^d^				
Team size^e^	Standard	35.4	56.6	0.01
	Large	64.6	43.4	
Disaster type	Natural disaster	60.9	56.4	0.58
	Man-made disaster	39.1	43.6	
Post-disaster phase^f^	< 1 week	18.8	16.9	0.003
	1–3 weeks	39.1	15.6	
	≥ 3 weeks	42.0	67.5	
Mission term^g^	< 1 month	39.1	24.4	0.05
	≥ 1 month	60.9	75.6	

Abbreviations: RN, registered nurse; LPN, licensed practical nurse; HA/DR, Humanitarian aid and disaster relief. Data are expressed as the mean (standard deviation) or percentage. The numbers vary due to missing data; the number of missing data is 1 for "post-disaster phase", 2 for "age", 3 for "years of experience in nursing" and "disaster medical training experience", and 6 for "team size". n = 147

^a^ The subjects were divided into two groups by nursing activity score (median score): < 94, Low; ≥ 94, High.

^b^ According to *t* tests (for continuous variables) or chi-square tests (for categorical variables).

^c^ Past experience before the recent experience of dispatch for HA/DR

^d^ Situation at dispatch for HA/DR

^e^ Team size was categorized into two groups by number of health professionals (< 24, standard; or ≥ 24, large).

^f^ Time from disaster occurrence to day of dispatch in weeks.

^g^ Length of dispatch to the disaster site.

Nursing activity scores are shown in Table 2. Median value of total scores, which indicate implementation frequency, was 94. Total score range was 45 to 145 points.

**Table 2:** Nursing activity score for each item.

Items	Nursing Activity Score	
	Mean	SD
Taking care of patients with internal diseases	3.5	1.2
Statistical analysis of patient data in the medical dispensary	3.3	1.4
Health management of JGSDF personnel	3.3	1.3
Management of the medical records of patients	3.2	1.5
Medical interview and history of patients	3.2	1.4
Efficient storage and consignment and of medical equipment in the dispensary	3.2	1.3
Maintenance of medical equipment	3.2	1.3
Disinfection of a variety of equipment	3.1	1.3
Explanation to patients about the drugs	3.0	1.4
Management of medical drugs	3.0	1.3
Maintenance of life support equipment	2.9	1.4
Management of the medical waste	2.9	1.3
Taking care of patients with skin conditions	2.9	1.2
Management of medical personnel’s working shift and job description	2.8	1.5
Use of the communication equipment	2.7	1.4
Wound irrigation or assistance with wound irrigation	2.7	1.2
Mental healthcare for JGSDF personnel	2.6	1.3
Care of patients with infection	2.5	1.2
Vaccination for local residents	2.3	1.4
Application of medical dressings or assistance with medical dressing	2.3	1.2
Care of patients with eye problems	2.2	0.9
Care of pediatric patients	2.1	1.4
Assessment of medical needs in the disaster site	2.0	1.2
Liaison and coordination with relevant organizations in Japan	2.0	1.2
Care of otological patients	2.0	0.9
Surgical debridement or assistance with surgical debridement	1.9	1.1
Medical education for local staff members	1.8	1.2
Dealing with illegal intruders	1.8	1.2
Small incisions or assistance with small incisions	1.8	1.0
Suturing or assistance with suturing	1.7	1.0
Suture removal or assistance with suture removal	1.7	1.0
Liaison and coordination with international organizations	1.6	1.1
Provision of health education to local residents	1.6	1.0
Screening of infectious diseases	1.6	1.0
Responding to the media	1.5	0.8
Care of expectant or nursing mothers	1.5	0.8
Mass-casualty triage	1.4	1.0
Cast immobilization or assistance with cast immobilization	1.4	0.8
Nutritional assessment	1.4	0.7
Water examination	1.3	0.8
Care of neonates	1.3	0.7
Surgeries or assistance with surgeries	1.3	0.6

Abbreviations: SD, standard deviation; JGSDF, Japan Ground Self-Defense Force. n = 147

A mean nursing activity score of 3.0 or above was seen in 10 of the 42 nursing activities. These 10 nursing activities were performed at a high frequency, with the three most frequent being “Taking care of patients with internal diseases”, “Statistical analysis of the patient data in the medical dispensary” and “Health management of JGSDF personnel”. Next, we added the mean and standard deviation (SD); 27 nursing activities achieved a score of 3.0 or higher while 15 scored below 3.0.

As shown in Table 3, the nursing activity score had significant positive correlations with sex, nursing license, disaster medical training experience, HA/DR experience, team size and post-disaster phase. Strong positive relationships between variables were noted between sex and nursing license; age and years of experience in nursing; and disaster type, post-disaster phase and mission term (Table 3).

**Table 3:** Spearman's rank correlation coefficients for nursing activity score and variables.

	Nursing Activity Score	1	2	3	4	5	6	7	8	9
1 Sex (Female)	0.32***									
2 Age (years)	0.002	-0.15								
3 Nursing license (RN)	0.35***	0.87***	-0.11							
4 Years of experience in nursing (years)	0.06	0.01	0.72**	0.03						
5 Disaster medical training experience	0.30**	0.01	0.17	0.20*	0.06					
6 HA/DR experience	0.21*	0.06	0.22**	0.08	0.21*	0.26**				
7 Team size (Standard)	0.21*	0.01	-0.09	0.11	-0.05	0.11	0.12			
8 Disaster type (Man-made disaster)	0.05	-0.15	0.28**	-0.18*	0.26**	0.05	-0.22**	-0.05		
9 Post-disaster phase (weeks)	0.26*	<-0.01	0.19*	-0.05	0.17*	0.10	-0.06	0.24**	0.75***	
10 Mission term (month)	0.16	0.02	0.04	-0.05	0.12	-0.04	-0.14	0.26*	0.57***	0.67***

*p<0.05

**p<0.01

***p<0.001.

The number of missing data are provided in the footnote of Table 1.

Table 4 shows the results of logistic regression analysis of nursing activity score with associated factors as independent variables. According to an analysis which included all variables, three factors demonstrated a significant relationship with the nursing activity score (Table 4). RN license was significantly associated with nursing activity score. Adjusted odds ratio of nursing activity score tended to increase with increases in the categories of disaster medical training experience, HD/DR experience and post-disaster phase, except for years of experience in nursing. Moreover, disaster medical training experience ≥ twice and post-disaster phase ≥ 3 weeks were significantly associated with nursing activity score in comparison with other categories (OR 2.90, OR 8.77, respectively).

**Table 4:** Associations between nursing activity score and independent variables: multiple logistic regression analyses.

	n	OR	95% CI	*p* value^a^
Nursing license				
LPN	88	ref.		
RN	59	7.79	2.95–20.57	<0.001
Years of experience in nursing (years)				
≤ 10	41	ref.		
11–20	66	0.58	0.21–1.56	0.28
≥ 21	37	0.59	0.18–1.95	0.38
Team size (standard)				
Large	75	ref.		
Standard	66	1.16	0.47–2.86	0.74
Disaster medical training experience				
None	65	ref.		
Once	28	2.56	0.83–7.92	0.1
≥ twice	51	2.90	1.12–7.49	0.03
HA/DR experience				
None	108	ref.		
Once	25	2.83	0.92–8.71	0.07
≥ twice	14	7.46	0.79–70.43	0.08
Post-disaster phase				
< 1week	26	ref.		
1–3 weeks	39	2.1	0.7–6.28	0.19
≥ 3weeks	81	8.77	2.59–29.67	<0.001

Abbreviations: OR, Adjusted odds ratio; CI, 95% confidence interval; RN, registered nurse; LPN, licensed practical nurse; HA/DR, humanitarian aid and disaster relief.

^a^ P values were calculated using multiple logistic regression analyses using the nursing activity score (High = 1, Low = 0) as a dependent variable and each variable as an independent variable, with adjustment for nursing license, age, disaster medical training experience, HA/DR experience, team size, and post-disaster phase.

The number of missing data are provided in the footnote of Table 1.

## Discussion

We found that nurses engage in 27 nursing activities in HA/DR, of which 10 are implemented at a high frequency. Furthermore, we found that a high nursing activity score was significantly associated with RN license, post-disaster phase (three weeks or longer), and past experience of disaster medical training (twice or more). Notably, there was a trend toward higher adjusted odds ratio of nursing activity score with more disaster medical training experience, HD/DR experience and post-disaster phase. This study, the first to examine factors associated with nursing activities in HA/DR using data analysis of the frequency of nursing activities, will contribute to the design of evidence-based disaster medical training and improve the quality of nursing services to survivors of disasters.

The higher nursing activity score, the more useful nurses were in the disaster site. A significant association with nursing activity score was shown for RN license. This finding showed that a significant association with nursing activity score was evident in the level of nursing license. This result was consistent with our expectations, given the difference in the preparation and scope of nursing practice between RNs and LPNs. First, the two groups differ with regard to education prerequisites and length of study; LPNs undergo two years of nursing education after graduating from junior high school or later, whereas RNs have three or four years of nursing education after graduating from high school. Secondly, the nursing knowledge of RNs is broader and deeper than that of LPNs [20]. Thirdly, the ratio of RNs to LPNs in Japan is about three to one, whereas more than seventy five percent RNs and LPNs work in either hospitals or clinics. Furthermore, Japanese law requires that LPNs operate under the supervision of RNs or medical doctors [21]. We consider that this difference in the scope of roles and responsibilities between RNs and LPNs influenced the results. This result may suggest the presence of issues in Japanese nursing qualification, as suggested by the ongoing effort of the Japan Nursing Association to stop the nursing course for LPNs [22]. In any case, LPNs account for a large percentage of the JGSDF nursing workforce. Nursing managers responsible for disaster preparedness should fully facilitate the use of LPNs to enhance the nursing capacity of medical care in the JGSDF. The question of whether the frequency or range of nursing activities changes in accordance with the number of years of schooling or nursing experts warrants further investigation, such as by comparing Certified Nurse Specialists to RNs.

Prior experience in dealing with disasters is already known to impact disaster preparedness [23] and awareness [24]. However, very few studies have considered whether prior experience in HA/DR is associated with subsequent HA/DR nursing activities. This study showed that nurses who had HA/DR experience had a higher nursing activity score than those having no HA/DR experience. Experienced firefighters are capable of grasping the situations even when they face difficult conditions. They are able to use their experience to make rapid and appropriate decisions [25]. Therefore we assume nurses utilize their experiences to increase the frequency of nursing activities.

Previous studies have evaluated the effect of training in disaster medicine [13, 26–27] by comparison of pre- and post-training scores. To our knowledge, however, the impact of experience of disaster medicine training on medical support activities in the reality of disaster situations has not been investigated. Further, presentation of disaster management in most nursing schools is insufficient [28]; for example, Weiner et al [29] found that only about 50% of nurses in the 348 schools of nursing in the US received necessary disaster-related content. Furthermore, although Yamamoto [30] suggested that there is a great need for nurses to develop disaster nursing competency, disaster nursing is rarely provided in basic nursing education in Japan, despite its integration into the basic nursing education curricula in 2009. This study identified a significant correlation between disaster medical training experience and nursing activity score. Indeed, only about 45% of the nurses in this study received disaster medical training. Kako et al [31] state that further disaster education opportunities should be available as a part of continuing education for all nurses. Our results provide evidence for the further development of disaster medical education and training, and for the development of disaster core competencies. Development of these competencies for health care providers to date was done through a qualitative rather than quantitative process [14, 32–33]. In contrast, our study collected quantitative data of actual nursing activities conducted by nurses during HA/DR, and examined factors which have a positive influence on nursing activity. Our findings will contribute to the design of evidence-based disaster medical training that will improve the quality of nursing care provided by nurses to survivors. Incorporating 10 nursing activities which are performed at a high frequency in our study into disaster medical training program for nurses might further improve essential competency for HA/DR. It is important to evaluate whether the evidenced-based disaster medical program truly improve nursing competency for HA/DR.

Chinese nurses have provided surgical care for victims of earthquakes in their home country, commonly including debridement and dressing, bandaging, fixation, manual handling, and mass casualty triage [16, 34]. This trend is consistent with reports from international disaster response that surgical care is the most frequent procedure in the early phase of disaster at overseas disaster sites [3–6]. In contrast, more than 55% of our present subjects were dispatched in the post-disaster phase, more than 3 weeks after the disaster had occurred, so surgical care and mass casualty triage were not frequently performed. Instead, we found that “Taking care of patients with internal diseases” was the most frequently nurse activity. This result supports a previous finding that medical needs at disaster sites change with time after the occurrence of the disaster [35]. Additionally, we also found that the post-disaster phase (more than three weeks) was significantly and positively correlated with the nursing activity score. Yokoyama et. al [36] found that dispatch at an earlier post-disaster phase was associated with the post-dispatch health problem of nurses who were dispatched to areas affected by the Great East Japan Earthquake of 11 March 2011. However, despite the importance of the post-disaster phase in disaster medicine, few studies have examined whether this variable influences the international disaster response. Here, we found that the adjusted odds ratio of nursing activity score was higher with the progression of post-disaster phase. This finding implies that disaster managers at the national level who dispatch FMTs in international disaster response should change the proportion of nursing staff in an FMT in accordance with post-disaster phase, as well as with the nature of the mission and length of dispatch.

Indirect nursing activity relating to patient care in HA/DR, such as the efficient storage and consignment and of medical equipment in the dispensary, maintenance of medical equipment, disinfection of a variety of equipment, management of medical drugs, maintenance of life support equipment, and management of medical waste, were relatively frequently performed in HA/DR. Given that FMTs are expected to be self-sufficient on arrival at a sudden-onset disaster zone and to be able to operate within it [7], indirect nursing activity is an important consideration for HA/DR. However, few studies have reported on indirect nursing activity. One study suggested that nurses should undertake different roles in the period after a disaster, namely clinician, 23communicator, leader, and psychosocial supporter [37]. Our findings imply that nurses are required to meet a wide range of roles in international disaster response, in not only direct but also indirect patient care. The nurses with high nursing activity score were very productive. However, it might be difficult for all nurses to have sufficient experiences for HA/DR. Therefore evidenced-based disaster medical program plays an important role to compensate the lack of their experience. Our results suggest that nurses should be equipped with the multiple nursing skills necessary for HA/DR through disaster medical training and daily nursing care at medical facilities, and will contribute to the establishment of disaster training curricula for HA/DR by disaster nursing educators and managers.

The loss resulting from the destruction associated with disasters leads to the development of various levels of psychological trauma in survivors [38], and psychological crisis intervention in disaster sites is a frequent nursing activity [16, 34]. For example, mental and psychiatric illness were identified as major health care service concerns in disaster shelters, and mental health care has been identified as one of the most important issues in disaster-stricken areas [39]. Mental health knowledge is required to meet the psychological needs of disaster victims as well as to help colleagues [40]. Disaster education for nurses should include mental health as basic knowledge [40]. In contrast, the Inter-Agency Standing Committee guidelines on mental health and psychological support in emergency settings state that minimum responses under health services should include specific psychological and social considerations in the provision of general health care [7]. FMTs have a modest role to play in mental health and psychosocial support following sudden-onset disaster, due to the short-term and surgically focused response they require [7]. Because only a few cases of mental health care by Japanese nurses for affected people in overseas disaster settings have been recognized, we excluded mental health care for affected people from our study questionnaire items, but included it for JGSDF personnel. Nurses scheduled to provide mental health care to affected people in an HA/DR should closely study the religion, culture and language of the disaster area in advance of dispatch, because they must provide triage for the mental health needs of survivors [41].

## Limitations

The above findings should be interpreted with caution in light of the following limitations. First, the study subjects were Japanese, and our results might not be generalizable to nurses in other countries with different licensing systems. Second, the number of subjects was limited and most were male. In contrast, the gender ratio of Japanese nurses is strongly skewed to females, and our results might therefore have been subject to selection bias. Third, the 42 nursing activities selected might not reflect all of the nursing activities in HA/DR performed by nurses. For these reasons, the results of this study must be interpreted with caution.

## Conclusions

To our knowledge, this is the first study to examine factors associated with nursing activities in HA/DR, using data analysis of the frequency of nursing activities. We confirmed that nurses engaged in 27 nursing activities in HA/DR, of which 10 activities were conducted at a higher frequency. Further, we also found that the frequency of nursing activities in HA/DR was significantly associated with RN license, post-disaster phase, and disaster medical training experience. Our findings will contribute to the design of evidence-based disaster medical training and improve the quality of nursing activities for survivors of disasters. We strongly recommend that international nurse leaders prepare for future large-scale disasters by conducting regular disaster training and incorporate disaster nursing education into the curriculum for all nurses.

## Supporting Information

S1 File Dataset. (XLSX)
